# Considerations for Subgroup Analyses in Cluster-Randomized Trials Based on Aggregated Individual-Level Predictors

**DOI:** 10.1007/s11121-023-01606-1

**Published:** 2023-10-28

**Authors:** Brian D. Williamson, R. Yates Coley, Clarissa Hsu, Courtney E. McCracken, Andrea J. Cook

**Affiliations:** 1 Biostatistics Division, Kaiser Permanente Washington Health Research Institute, Seattle, WA USA; 2 Vaccine and Infectious Disease Division, Fred Hutchinson Cancer Center, Seattle, WA USA; 3 Department of Biostatistics, University of Washington, Seattle, WA USA; 4 Investigative Science Division, Kaiser Permanente Washington Health Research Institute, Seattle, WA USA; 5 Center for Research and Evaluation, Kaiser Permanente Georgia, Atlanta, GA USA

**Keywords:** Heterogeneity of treatment effects, Subgroup analyses, Cluster-randomized trials, Ecological studies, Health disparities

## Abstract

In research assessing the effect of an intervention or exposure, a key secondary objective often involves assessing differential effects of this intervention or exposure in subgroups of interest; this is often referred to as assessing effect modification or heterogeneity of treatment effects (HTE). Observed HTE can have important implications for policy, including intervention strategies (e.g., will some patients benefit more from intervention than others?) and prioritizing resources (e.g., to reduce observed health disparities). Analysis of HTE is well understood in studies where the independent unit is an individual. In contrast, in studies where the independent unit is a cluster (e.g., a hospital or school) and a cluster-level outcome is used in the analysis, it is less well understood how to proceed if the HTE analysis of interest involves an individual-level characteristic (e.g., self-reported race) that must be aggregated at the cluster level. Through simulations, we show that only individual-level models have power to detect HTE by individual-level variables; if outcomes must be defined at the cluster level, then there is often low power to detect HTE by the corresponding aggregated variables. We illustrate the challenges inherent to this type of analysis in a study assessing the effect of an intervention on increasing COVID-19 booster vaccination rates at long-term care centers.

## Introduction

A key secondary objective in many clinical or epidemiological studies is to determine whether an intervention has a differential effect across subgroups of study participants, also referred to as effect modification (see, e.g., Pearce & Greenland, 2005; VanderWeele & Robins, 2007; Greenland, 2009) or heterogeneity of treatment effects (HTE) (Willke et al., 2012; Varadhan & Seeger, 2013; Angus & Chang, 2021). We adopt the latter term in this article, to avoid implying a causal or biological interpretation of the interaction between intervention and subgroup characteristic. If the intervention effect is heterogeneous, then resources can be prioritized to maximize benefit and reduce possible risks (Knol & VanderWeele, 2012; Rothwell, 2005). This analytical approach can also be used to advance health equity by identifying interventions that may remediate or exacerbate health disparities (Cintron et al., 2022; Petkovic et al., 2020; Ward et al., 2019). In response, the design or deployment of such “intervention-generating interventions” may be adapted to ensure equitable benefit (Veinot et al., 2018). For example, a recent study assessing a financial incentives intervention to improve the uptake of colorectal cancer screening showed improvement overall, but, more importantly, reduced health disparities by increasing colorectal cancer screening rates in previously under-screened racial and ethnic groups to a greater extent than those with higher baseline screening rates. As a result, colorectal screening rates were almost equal across race and ethnicity groups in the intervention arm; in the control arm, large screening disparities were observed across race and ethnicity groups (Green et al., 2019).

The COVID-19 pandemic has disproportionately affected individuals who work in health care, including at long-term care communities (LTCCs). Nursing home staff and residents have experienced higher rates of COVID-19 cases and deaths throughout the pandemic (Chidambaram, 2022). At the same time, there has been a lag in COVID-19 vaccinations among people working at LTCCs. LTCC staff are often low-wage workers who come from diverse social, cultural, racial, and ethnic backgrounds (Scales, 2020; True et al., 2020). The Engaging Staff to Improve COVID-19 Vaccination Response at Long-term Care Facilities (ENSPIRE) study (NCT05449418, 2023), funded by the Patient-Centered Outcomes Research Institute (PCORI), is a cluster-randomized trial testing the comparative effectiveness of COVID-19 booster shot promotional materials tailored to specific racial and ethnic groups compared to more generic materials among LTCC staff in two states in the United States—Georgia and Washington. The ENSPIRE study was designed to test whether more customized materials co-designed by LTCC staff from similar backgrounds increases booster vaccination in this at-risk population (PCORI, 2021). Co-design processes have been used in several studies to improve health-related outcomes (Norman et al., 2013; Westfall et al., 2016; Schoeppe et al., 2017; Jackson et al., 2022). The primary outcomes in ENSPIRE are the aggregate COVID-19 booster rate at each participating LTCC and a COVID-19 booster Net Promoter Score (NPS) collected using a staff survey. Several HTE analyses were also proposed to understand the impact of the co-design intervention in different subgroups of LTCC staff. Specifically, we proposed to evaluate the impact of region (Georgia vs. Washington), baseline booster uptake (above/below median site-level booster rate), and geography (urban/suburban vs. rural). While conducting HTE analyses based on self-reported race and ethnicity aggregated at the LTCC level would have been enlightening and relevant given the focus of the study, neither the sites nor any other entity had accurate race or ethnicity data for all staff at participating sites. Given the lack of data we had to forego conducting an HTE analysis based on this variable. The research presented in this paper was motivated by our planning for the ENSPIRE trial as we considered how we would conduct statistically sound HTE analyses by race and ethnicity if such data were available.

Statistical approaches to assessing HTE often involve modeling interactions between variables (Greenland, 2009). In cases with individual-level outcomes and possible HTE, this is well-understood; however, power for detecting HTE is often much lower than for detecting an intervention effect (see, e.g., Greenland, 1993). Assessing HTE with cluster-level outcomes and aggregated individual-level variables is less well understood (Petkovic et al., 2020). Data are only available at the aggregate setting in many public health settings, like the ENSPIRE study; studies using such data are often referred to as ecological studies (Wakefield, 2008). In the literature on such studies, the term *ecological bias* describes the phenomenon that individual-level effects—typically associations—are often not identifiable based on aggregate data (Wakefield, 2008). Greenland and Morgenstern (1989) show examples where ecological bias may be present for the association between the exposure and outcome of interest due to either aggregated confounders or aggregated variables for which there is observed HTE. In randomized studies, it is often necessary to adjust the final analysis for baseline variables that were used in randomization (Li et al., 2017), and in some cases, adjusting for other variables can improve precision (Tsiatis et al., 2008; Ye et al., 2022). However, including aggregated variables for which HTE may be present may introduce bias.

Using aggregate variables is further complicated in settings where the variable of interest is typically modelled using a collection of mutually exclusive binary indicator variables. For example, suppose that self-reported race may be one of “White,” “Black/African American,” “Asian,” or “All other races.” Binary indicators could then be created; for example, for each category besides “White”, a binary indicator that the self-reported race was that category. If aggregated to the proportion level, these variables (e.g., “proportion identifying as Black/African American”) are *compositional data* (Aitchison, 1982): in other words, changing the value of one proportion necessarily changes at least one of the others, rendering interpretation of regression coefficients more difficult than in the individual-level data case (Lu et al., 2019).

In this manuscript, we investigate challenges in assessing HTE based on aggregated individual-level variables in cluster-randomized trials. In the next section, we describe the ENSPIRE study, including planned subgroup analyses and the rationale for not performing subgroup analyses based on an aggregated version of self-reported race. In the “Methods” section, we describe a hypothetical individual-level data structure that could lead to several cluster-level data structures and discuss the interpretation of regression coefficients fit to each level of data. In the “Results” section, we generate data mimicking possible distributions of covariates and outcomes for ENSPIRE study participants and LTCCs and matching this hypothetical data structure and present results from trying to assess HTE by self-reported race. Additional results can be found in the Supporting Information. Code to reproduce all results is available on GitHub at https://github.com/bdwilliamson/cluster_subgroup_analyses_supplementary.

## The ENSPIRE Study: Engaging Staff to Improve COVID-19 Vaccination Response at Long-Term Care Facilities

The ENSPIRE study enrolled 40 LTCCs with approximately 4000 long-term health care workers in two geographically, culturally, and ethnically diverse states within the United States (US) from May through July, 2022. Eligibility criteria for LTCCs were (i) physically located in the state of Georgia (Ga.) or Washington (Wash.), (ii) approximately 50–120 individuals on staff, and (iii) COVID-19 staff booster vaccination rate 60% or less as of December 1, 2021. Efforts were made to recruit both urban/suburban and rural locations across the two states, where urban/suburban is defined as a ZIP code within the Atlanta or Seattle metropolitan statistical areas (Ga. and Wash., respectively). All staff members at participating LTCCs were invited to participate in a staff survey, which collected self-reported information on demographic characteristics, COVID-19 vaccination history, and attitudes about vaccines, including a Net Promoter Score (NPS) measuring willingness to recommend a COVID-19 booster vaccine to a co-worker. The NPS is measured using a 10-point Likert scale, with 10 meaning most likely to recommend the booster; we created a binary COVID-19 booster promoter outcome by labeling NPS ≥ 9 as a “promoter” (Piraux et al., 2022).

Race and ethnicity were measured at a granular level using a single question on the staff survey, allowing participants to select specific cultural identities from among 57 total options. Because tailoring vaccine messages to a specific cultural group was integral to ENSPIRE, it was critical that the race, ethnicity, and culture of the staff at the LTCCs in the study be measured at a granular level. The approach focused more on culture and country of origin rather than the historical US census classifications. Thirteen possible categories were chosen: White, Native Hawaiian or Pacific Islander, American Indian or Alaska Native, Hispanic, African, African American/Black, Afro-Caribbean, Asian, South Asian, Middle Eastern or North African, multi-racial/multi-cultural, not specified (with fill in the blank), and a prefer not to answer option. Using survey branching logic, survey respondents could select additional options for some of the categories listed above. For example, respondents selecting African were then prompted to select an additional cultural designation which included options such as: Nigerian, Ethiopian, Ghanaian, South African, and Somalian. These additional cultural designations were selected by the study team based on the prevalence rates of these cultures in the United States. Participants could select more than one option. In total, 57 racial, ethnic and/or cultural designations were provided.

When the study was designed, it was determined that individual-level COVID-19 vaccination data would be difficult to collect and could not be successfully collected from a representative number of people employed at LTCCs. At the same time, through federal and state mandates, aggregate-level vaccination data were being collected by all sites in Washington state and the vast majority of LTCCs in Georgia. These data were collected on all staff members eligible to work in a given week and were made publicly available through the US Centers for Medicare and Medicaid Services (CMS, 2022). For most enrolled sites, these data could still be obtained by the study team even if a LTCC withdrew from the study, reducing the likelihood of missing outcome data. The sample size of 40 LTCCs for ENSPIRE was determined based on power calculations designed to ensure at least 90% power to detect an increase of at least 13.3% in booster vaccination rates comparing the intervention arm to the usual care arm. We assumed an average of 100 staff members per LTCC; if only one-third of staff members at each site would complete both the baseline and follow-up surveys, this provides at least 90% power to detect a difference in NPS promoter rate of 10.3% between arms, assuming intra-class correlation of 0.01. The LTCCs were randomized to one of two intervention conditions using constrained randomization (Moulton, 2004), stratified by state and balancing on staff COVID-19 booster vaccination rates (above vs. below median in the state), rural vs. urban/suburban location, baseline staff survey response rate (above vs. below median in the state), and number of staff members (above vs. below median in the state). The trial intervention conditions are enhanced usual care (COVID-19 vaccine promotion materials from the US Centers for Disease Control and Prevention or other national organization) and a co-design intervention with peer advocacy training.

A key aspect of the co-design intervention is the recruitment of LTCC staff members to design tailored COVID-19 booster vaccination promotion materials. Co-design efforts will focus on language, cultural, and ethnic affinity groups in each state. The goal is to identify 4 affinity groups per state and recruit one staff member per identified affinity group from each of the LTCCs randomized to the co-design intervention arm to participate in co-design workshops. The affinity groups are to be selected based on ethnic or other key demographic characteristics (such as language and/or age) that might impact individuals’ perspectives and values around COVID-19 vaccination. Through the co-design intervention, we seek to affect both individual and community attitudes towards COVID-19 booster vaccines. The two primary outcomes, individual-level NPS score and center-level COVID-19 booster vaccination rate, are designed to provide information at both levels. Outcome data is collected at three timepoints, beginning at 2 months after the intervention materials were mailed to the LTCCs and spaced at 2-month intervals. The final outcome data collection timepoint is in August 2023.

In the primary analysis for ENSPIRE, we will fit two generalized estimating equations (GEE) regression models (Liang & Zeger, 1986). For the first primary outcome, staff COVID-19 booster vaccination rate at a LTCC, we will fit a GEE linear regression. For the second primary outcome, individual COVID-19 booster Net Promoter Score (NPS), we will fit a GEE logistic regression using as outcome the binary promoter outcome (NPS ≥ 9). The primary models will include adjustment for constrained randomization factors but will not include adjustment for the aggregated version of self-reported race, as we discussed in the “Introduction” section. HTE by pre-specified variables will be assessed by fitting further interaction models.

As we discussed in the “Introduction” section, we plan to analyze HTE based on several variables but chose not to consider an aggregated version of self-reported race. All three planned analyses are based on variables with complete and reliable capture: region (Georgia vs. Washington), baseline booster uptake (above vs. below median in the state), and urban/suburban vs. rural location. While considering HTE based on self-reported race is important, several factors led us not to consider it in this case. First, self-reported race is only assessed through the staff survey, which we expect will only be completed by a fraction of eligible staff at each site. This complicates the interpretation of an aggregated version of self-reported race, as it may not be representative of the LTCC, and was our primary reason for not assessing HTE by self-reported race or ethnicity. Second, we provided many choices of race and ethnicity categories. This complicates the interpretation of an HTE analysis since the multiple race and ethnicity categories create compositional data at the LTCC level.

In the next section, we describe a simplified data structure at the individual level that could give rise to a cluster-level dataset based on aggregating certain variables. We discuss the interpretation of regression coefficients in these models through several examples, including urban/suburban vs. rural status and self-reported race in a scenario where there are only two race categories to choose from.

## Methods

### Data Structure

We assume that each study participant (staff member) $i$ ($i=1, \dots , {n}_{c}$) in cluster $c$ ($c=1, \dots , K$) has data on the following characteristics: indicator of intervention assignment ${A}_{ci}$, where ${A}_{ci}=1$ if cluster $c$ was randomized into the intervention arm, and ${A}_{ci}=0$ otherwise; subgroup variable(s) of interest ${Z}_{ci}$, for example, ${Z}_{ci}={Z}_{c}=1$ if the long-term care center is in an urban/suburban location vs. ${Z}_{c}=0$ if the center is in a rural location; a vector of other baseline covariates ${W}_{ci}$; and a follow-up binary outcome ${Y}_{ci}$, e.g., ${Y}_{ci}=1$ if participant $i$ within center $c$ received a booster vaccine and zero otherwise. Considering only a single timepoint for outcome data collection simplifies the analysis strategy that we employ. Thus, the individual-level data consist of $(A, Z, W, Y)$.

In some cases, only data at the cluster level are available. In this scenario, ${\overline{Y} }_{c}= \frac{1}{{n}_{c}}{\sum }_{i=1}^{{n}_{c}}{Y}_{ci}$ is the observed cluster-level outcome, defined as the mean outcome value at cluster $c$; for example, in the ENSPIRE study, only ${\overline{Y} }_{c}$ is obtained and represents the proportion of staff who have received a booster vaccine at long-term care center $c$. The indicator of intervention assignment ${A}_{c}$ is easily rolled up to the cluster level, since we consider a cluster-randomized trial (i.e., ${A}_{ci}=a$ for all participants in cluster $c$). Finally, we consider cluster-level versions of the subgroup variables and other covariates. Some variables (e.g., urban/suburban vs. rural location of the center) are defined at the cluster level. Variables that are instead defined at the individual level must be aggregated in some way. For example, if there are $p$ individual-level covariates $W$, we could consider cluster-level versions $\left({X}_{c1},\dots , {X}_{cp}\right)$, where ${X}_{cj}= \frac{1}{{n}_{c}}{\sum }_{i=1}^{{n}_{c}}{W}_{cij}$ for $j=1, \dots , p$. The cluster-level data consist of $(A, U, X, \overline{Y }$), where $U$ is the (possibly aggregated) subgroup variable(s) of interest and $X$ is the (possibly aggregated) other baseline covariates.

Given the available data at either the individual or cluster level, one may choose an appropriate analysis technique. Next, we summarize several different regression models that may be used for each data type described above.

### Individual-Level Models

For situations where individual-level data are available, we consider linear regression models on the binary outcome ${Y}_{ci}$ to estimate the probability of having received a booster vaccine. There are two quantities of interest: the risk difference between the intervention and control arms and the difference in this risk difference based on levels of a subgroup variable (i.e., HTE) achieved by including the appropriate interaction terms in the model. For example, HTE by a single binary variable ${Z}_{cij}$ could be defined using the following mean model:

$$E\left({Y}_{ci}\right|{A}_{ci}=a, {Z}_{cij}=z, {W}_{ci}=w)= {\beta }_{0}+ {\beta }_{1}a+ {\beta }_{2}z+ {\beta }_{3}az+{\beta }_{w}w.$$

In this model, ${\beta }_{3}$ is interpreted as the mean difference in the risk difference of receiving a booster vaccine between the intervention and control arms for those in subgroup ${Z}_{cij}=1$ relative to those in subgroup ${Z}_{cij}=0$.

To estimate ${\beta }_{3}$, we fit generalized estimating equations (GEE) models assuming an identity link function and normal error structure and account for cluster randomization using a sandwich variance estimate with an independent working correlation structure (Liang & Zeger, 1986). We chose to use a linear regression model over a logistic regression model (which is often used in situations with binary outcomes) for two reasons. The first is that our primary quantity of interest is the risk difference of receiving a booster vaccine in the intervention arm compared to the control arm; ${\beta }_{3}$ in the linear regression model has this interpretation, which is useful in our context when disseminating results. If we had instead used logistic regression, the interpretation of regression parameters would be as a difference in the log-odds of receiving a booster vaccine. The second reason for choosing a linear regression model over a logistic regression model is to achieve a similar interpretation between the individual- and cluster-level models: in both cases, we consider a mean difference. A logistic regression model would not be a default choice in the cluster-level case with an outcome defined as a proportion, though such a model can be fit if the number of successes and failures are known. It is possible that a logistic regression model could have different operating characteristics for estimating the difference in booster vaccination rates (e.g., type I error and power), though we expect the trends in these operating characteristics to be similar between the two estimation procedures. A possible downside of choosing a linear regression model is that predicted values can fall outside of [0, 1]; since our goal is to estimate a risk difference and not to make predictions, we judged this an acceptable tradeoff.

### Cluster-Level Models

When only cluster-level data are available, the cluster-level outcome ${\overline{Y} }_{c}$ is the mean individual-level outcome value. We consider linear regression to model this continuous outcome of the mean cluster-level proportion having received a booster vaccine. As in the individual-level case, there are two quantities of interest: the mean difference in the proportion having received the booster vaccine between the intervention and control arms and the difference in this mean difference based on levels of a subgroup variable; the latter is estimated by including the appropriate interaction terms in the model. For example, HTE by a single subgroup variable ${U}_{cj}$ could be defined using the following mean model:

$$E\left({\overline{Y} }_{c}\right|{A}_{c}=a, {U}_{cj}=u, {X}_{c}=x)= {\alpha }_{0}+ {\alpha }_{1}a+ {\alpha }_{2}u+ {\alpha }_{3}au+ {\alpha }_{x}x.$$

In this model, ${\alpha }_{3}$ is interpreted as the mean difference in the mean difference between the proportion having received a booster vaccine at intervention versus control clusters for each one-unit difference in the subgroup variable ${U}_{cj}$. For example, if ${U}_{cj}=1$ if center $c$ is in an urban/suburban location and 0 if center $c$ is in a rural location, then the HTE term is relatively easy to interpret: a one-unit difference in ${U}_{cj}$ compares centers in urban/suburban locations to those in rural locations. It follows that ${\alpha }_{3}$ quantifies the difference in the intervention effect on vaccination rates between urban/suburban and rural centers.

However, interpretation of this mean difference in differences is particularly challenging if the subgroup variable is naturally defined at the individual level and not the cluster level. In ENSPIRE, as in other studies, it is important to determine if intervention effects differ by self-reported racial category; this can aid in prioritizing resources for historically underserved communities to ensure that everyone benefits from an effective intervention. We could consider in this case an individual-level subgroup variable ${Z}_{cij}$, where ${Z}_{cij}=1$ if participant $i$ within cluster $c$ identifies as Black/African American and 0 otherwise. We could define an aggregated, cluster-level version of this variable in several ways. Two that we have considered are (i) using ${U}_{cj}= {V}_{cj}- \frac{1}{K}{\sum }_{c=1}^{K}{V}_{cj}$ where ${V}_{cj}= \frac{1}{{n}_{c}}{\sum }_{i=1}^{{n}_{c}}{Z}_{cij}$, i.e., ${V}_{cj}$ is the proportion of staff at center $c$ identifying as Black/African American and ${U}_{cj}$ is a mean-centered version of this proportion; or (ii) using ${U}_{cj}= I\left(\frac{1}{{n}_{c}}{\sum }_{i=1}^{{n}_{c}}{Z}_{cij}>t\right)$, i.e., the binary indicator that the proportion of staff at center $c$ identifying as Black/African American is larger than $t$, which could be used to define “high” versus “low” proportions. If using approach (i) above, care must be taken to center the variable around the mean proportion in the study so that the appropriate regression coefficients can be interpreted as differences from this mean level. Additionally, as mentioned in the Introduction, interpreting such an effect is complicated if the proportions define a compositional variable.

To estimate ${\alpha }_{3}$, we can use the same GEE approach that we used for the individual-level models, which is equivalent in this case to using ordinary least squares with robust standard error estimators (Lipsitz et al., 1994).

In the next section, we evaluate the performance of these different approaches using a simulation study.

## Methods for Simulating Data Mimicking ENSPIRE

We now detail our specific procedure for simulating data to evaluate performance of the approaches outlined above. In all cases, we simulate data that mimic ENSPIRE but in a simpler setting: there are only two possible categories of self-reported race, Black/African American and all other races. We simulate data at the individual level by first sampling $K\in \{30, 40, 80\}$ centers within one of two regions (${R}_{c}\in \{0, 1\}$ for each $c=1, \dots , K$) and from either urban/suburban or rural areas. We set all centers to have 100 staff members (i.e., ${n}_{c}=100$ for $c=1, \dots , K)$. These staff members each have a self-reported race. Next, we randomize the centers to intervention or usual care (control) using constrained randomization stratified by region and attempting to balance on urban/suburban vs. rural status (Li et al., 2017). Finally, we obtain individual-level booster vaccination data by modeling the probability of receiving a booster vaccine conditional on region, urban/suburban vs. rural location, self-reported race, and intervention assignment as following a linear regression model with known true regression coefficients. In this scenario, the individual-level data consists of $(A, Z, W, Y)$, where ${A}_{ci}$ is the observed intervention assignment; ${Z}_{ci}=({Z}_{ci1},{Z}_{ci2})$, ${Z}_{ci1}=1$ if center $c$ is in an urban/suburban location and 0 otherwise, and ${Z}_{ci2}=1$ if participant $i$ at center $c$ identifies as Black/African American and 0 otherwise; ${W}_{ci}={R}_{c}$; and ${Y}_{ci}=1$ if participant $i$ at center $c$ receives a booster vaccine. The cluster-level data consists of $(A, U, X, \overline{Y })$, where ${A}_{c}$ is the observed intervention assignment; ${U}_{c}=({U}_{c1},{U}_{c2})$, where ${U}_{c1}=1$ if center $c$ is in an urban/suburban location and 0 otherwise, and ${U}_{c2}$ is an aggregated version of the self-reported race variable; ${X}_{c}={R}_{c}$; and ${\overline{Y} }_{c}$ is the booster vaccination rate at center $c$. We consider two versions of ${U}_{c2}$: the proportion of staff members identifying as Black/African American, and an indicator of whether this proportion is greater than 0.5. The precise definitions of each variable and the true values of each regression coefficient are provided in the Supporting Information.

The values of the true regression coefficients define nine scenarios, which are displayed in Table 1. In all cases without HTE, the intervention is associated with a 14% difference in the probability of receiving a booster vaccine, where individuals at centers in the intervention arm tend to have higher probabilities; urban/suburban status is associated with a 3% difference in the probability of receiving a booster vaccine, where individuals at urban/suburban centers tend to have higher probabilities, and self-reported race is associated with a 1% difference in the probability of receiving a booster vaccine, where people who identify as Black/African American tend to have a higher probability of receiving a booster vaccine. When present, HTE by urban/suburban vs. rural status results in individuals at urban/suburban centers having a 10% larger difference in probabilities of receiving a booster vaccine between intervention and control compared to individuals at rural centers. When present, HTE by self-reported race results in individuals identifying as Black/African American having a 15% larger difference in probabilities of receiving a booster vaccine between intervention and control compared to individuals not identifying as Black/African American. These true regression coefficients were chosen with three considerations in mind: (i) the true intervention effect with no HTE should be the same as in the ENSPIRE power calculations, (ii) the true HTE by self-reported race should be large enough to detect with a modest sample size using an individual-level model, and (iii) the combination of parameter values should result in valid datasets that could be randomized using our chosen constrained randomization software and analyzed using our chosen regression software. The values in Table 1 satisfy these requirements, while the scenarios allow us to explore the operating characteristics of estimators of each parameter.

**Table 1:** Scenarios for data generation based on which variables result in differences in the probability of receiving a booster vaccine or result in treatment effect heterogeneity (HTE) (i.e., change the effect of intervention on the probability of receiving a booster vaccine)

Scenario	Difference in probability of receiving booster vaccine by urban/suburban vs. rural status	Difference in probability of receiving booster vaccine by self-reported race	HTE by urban/suburban vs. rural status	HTE by self-reported race
1				
2	✔			
3		✔		
4	✔	✔		
5	✔		✔	
6		✔		✔
7	✔	✔	✔	
8	✔	✔		✔
9	✔	✔	✔	✔

For all scenarios, we fit regression models at both the individual and cluster level that adjusted for differing sets of variables (see the “Methods” section for model framework). The regression models are specified in Table 2; all models include the intervention assignment and adjustment for the randomization factors (region and urban/suburban vs. rural status). We use a small-sample correction for the variance estimator for all parameters, defined as $K/ (K-2)$, since we used two variables in constrained randomization (Fay & Graubard, 2001).

**Table 2:** Regression models fit for each scenario specified in Table 1. All models include the intervention assignment and adjustment for randomization factors (region and urban/suburban vs. rural). For the center-level models that include self-reported race, the variable used to encode self-reported race at the cluster level is displayed in parentheses

Level of data	Model	Adjusted for self-reported race	Allows HTE between intervention and urban	Allows HTE between intervention and self-reported race
	1a			
	1b		✔	
Individual	1c	✔		
	1d	✔	✔	
	1e	✔		✔
	1f	✔	✔	✔
	2a			
	2b		✔	
	2c	✔(proportion)		
	2d	✔(indicator)		
Center	2e	✔(proportion)	✔	
	2f	✔(indicator)	✔	
	2 g	✔(proportion)		✔
	2 h	✔(indicator)		✔
	2i	✔(proportion)	✔	✔
	2j	✔(indicator)	✔	✔

For each scenario and number of clusters $K\in \{30, 40, 80\}$, we generated 1000 random datasets according to the specification outlined above and fit each model in Table 2 to each dataset. Based on the estimated regression coefficients and their associated sandwich standard errors, we implemented a test of the null hypothesis for each regression parameter, with a focus on testing the null hypothesis that there is no difference in the intervention effect between those who identify as Black/African American and those who do not. The code necessary to reproduce all simulation results is available on GitHub.

## Results

### Main Simulation Results

**Fig. 1:**
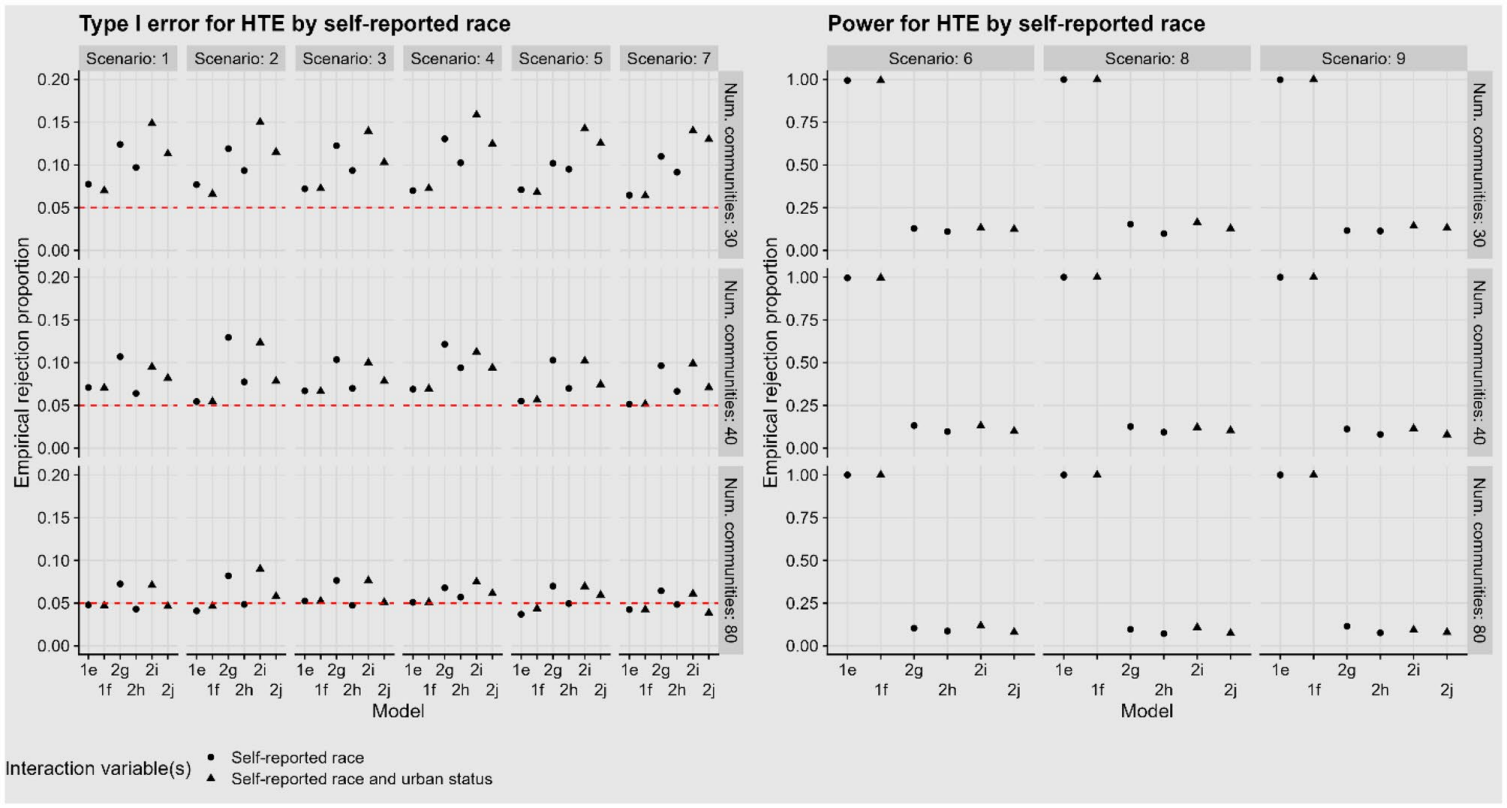
Proportion of tests rejected, where the null hypothesis is no difference in the effect of intervention between people who identify as Black/African American vs. those who do not. Left: type I error (scenarios with truly no HTE). Right: power (scenarios with true HTE). The number of centers increases down the rows, while the differing scenarios are shown in the columns. The models correspond to: individual-level models including an interaction between self-reported race and intervention assignment (1e) and also between urban/suburban vs. rural and intervention assignment (1f); cluster-level models using the proportion identifying as Black/African American, including an interaction between self-reported race and treatment (2g) and also between urban/suburban vs. rural and intervention assignment (2i); and cluster-level models using the indicator that the proportion identifying as Black/African American is greater than 0.5, including an interaction between self-reported race and intervention assignment (2h) and also between urban/suburban vs. rural and intervention assignment (2j)

Type I error for testing for HTE comparing people who do or do not identify as Black/African American is controlled in the simplest setting when using individual-level models (Fig. 1). These models also result in uniformly high power to detect HTE. In contrast, the cluster-level models tend to have higher type I error and have uniformly low power, with highest type I error and lowest power in the cases where the continuous proportion is used at the cluster level.

### Additional Simulation Results

We provide further results for both regression parameter estimation and hypothesis testing under the simplest set of scenarios studied in the previous subsection in the Supporting Information. The individual-level models consistently had the best performance, in terms of bias for estimators of regression parameters and type I error and power for hypothesis tests. In cases where there truly was no HTE, it often was beneficial not to include parameters for HTE in the regression models (see, e.g., Figure S1). The cluster-level models had varying performance: aggregating the self-reported race variable led in many cases to poor type I error control and power for all hypothesis tests, with the worst performance observed when the cluster-level proportion was used.

We also considered several ways to make the data-generating mechanism more realistic: adding heterogeneity in the distribution of urban status, adding heterogeneity in the distribution of self-reported race, and allowing some study participants to have already received a booster vaccine at baseline. A full set of results under all combinations of these corresponding scenarios are provided in the Supporting Information, including bias for estimators of regression parameters and type I error and power for hypothesis testing.

**Fig. 2:**
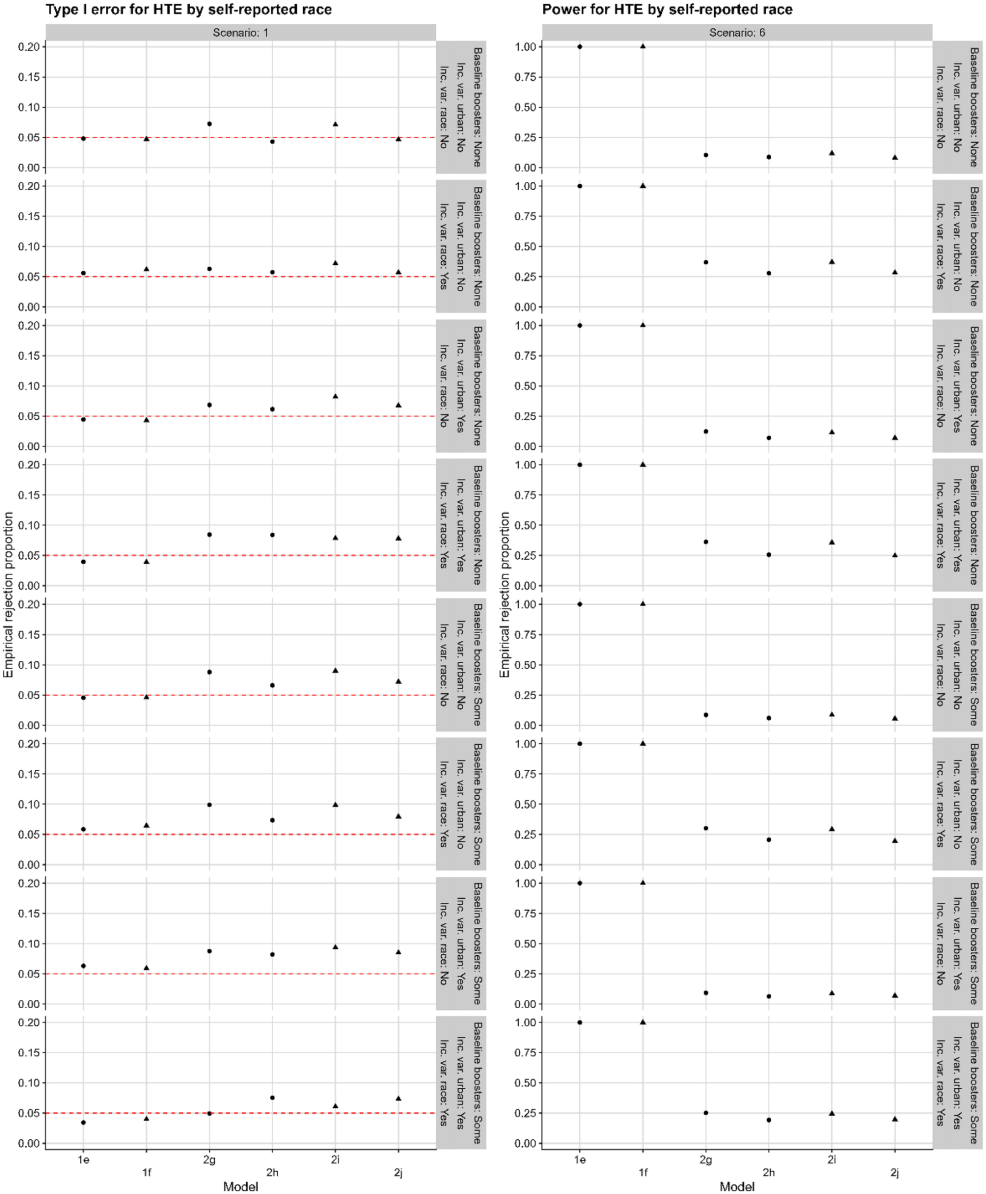
Proportion of tests rejected in a case with 80 centers, where the null hypothesis is no difference in the effect of intervention between people who identify as Black/African American vs. those who do not. Left: type I error (under Scenario 1 from Table 1, with truly no HTE). Right: power (under Scenario 6 from Table 1, with true HTE). The rows display all possible combinations of allowing some participants to have received the booster vaccine at baseline and allowing heterogeneity in the distribution of urban status and self-reported race. The models correspond to individual-level models including an interaction between self-reported race and intervention assignment (1e) and also between urban/suburban vs. rural and intervention assignment (1f); cluster-level models using the proportion identifying as Black/African American, including an interaction between self-reported race and treatment (2g) and also between urban/suburban vs. rural and intervention assignment (2i); and cluster-level models using the indicator that the proportion identifying as Black/African American is greater than 0.5, including an interaction between self-reported race and intervention assignment (h) and also between urban/suburban vs. rural and intervention assignment (2j)

In Fig. 2, we show type I error and power for testing for HTE comparing people who do or do not identify as Black/African American under all possible combinations of the more complicated data-generating mechanisms. For simplicity, we restrict to 80 centers and only consider scenarios 1 (the simplest scenario with no HTE) and 6 (the simplest scenario with HTE by self-reported race). The results are consistent with those observed in the simplest setting presented above: the individual-level models achieve type I error control and high power, while the cluster-level models have uniformly lower power.

Adding heterogeneity in the distribution of urban status mainly affected type I error for the test of a difference in booster vaccination rate between intervention arms by urban status (see, e.g., Figure S84). Type I error for HTE by urban status was worse in small samples but better in large samples, while type I error for HTE by self-reported race was inflated (see, e.g., Figure S108). Power and bias were similar for both parameters (see, e.g., Figures S85 and S109).

Adding heterogeneity in the distribution of self-reported race affected many results. Power for rejecting the null hypothesis of no intervention effect increased in the cases where it was initially low, with a corresponding reduction in bias (and the variability of the bias) (see, e.g., Figures S15—S17 compared to Figures S9—S11). Type I error for detecting a difference in booster vaccination probability by urban/suburban vs. rural status was inflated in many cases with no power gain (see, e.g., Figure S39); type I error for detecting a difference in booster vaccination probability by self-reported race was inflated in some cases but not in others, also with no power gain (see, e.g., Figure S63). Type I error decreased for urban HTE (see, e.g., Figure S84); type I error increased for HTE by self-reported race (see, e.g., Figure S111). In all cases, the distribution of bias was less variable (comparing, e.g., Figure S113 to S11). Adding people with baseline outcomes mainly affected the estimation of urban effects and HTE in the cluster-level analyses. We observed marked decreases in power and a slight increase in type I error in some scenarios (see, e.g., Figure S114).

## Discussion

The ENSPIRE study was designed to test an intervention to lower barriers to accessing COVID-19 vaccines in a population that has been disproportionately affected by the COVID-19 pandemic and where many people identify with groups that have historically been underrepresented in health research. Focusing on a cluster-level outcome provided reliable capture of the outcome of interest; however, this choice precluded assessing HTE by self-reported staff-level race and ethnicity. We found through our simulation study that even with many clusters (*N* = 80), which is likely infeasible in the context of this study, there is still low power to detect HTE by an aggregated version of self-reported race in the simple setting where there are only two possible racial categories to choose from.

While this setting is a simplification of the racial, ethnic, or cultural categories typically observed, it nonetheless suggests that the results might be more pronounced in cases with multiple categories (i.e., compositional data), which would result in an increased number of regression parameters and render the interpretation of these parameters (and resulting hypothesis tests) more difficult. The growing literature on incorporating intersectionality theory into public health research (Bauer, 2014; Glymour & Rudolph, 2016; Wemrell et al., 2017a, b), which has many advantages, also necessitates finer measurement of social identity, implying the aggregation of greater numbers of individual-level variables to the cluster level. In ENSPIRE, where there are 51 possible cultural identities, some form of coarsening is necessary to achieve enough staff members in each group; however, as we found in our simulation study, it still may be difficult to achieve adequate power to detect HTE regardless of the number of coarsened cultural identity groups.

It is possible that a meta-analysis approach could combine the results from multiple trials to assess HTE to improve power; however, many aspects of the design need to be harmonized across the constituent trials in the meta-analysis, including the possible racial categories and the way that self-reported race is aggregated to the cluster level. In contrast, individual-level analyses, where possible, provide power to detect HTE and are a key tool towards adapting interventions to reduce disparities.

Better estimation of HTE for populations that experience persistent health disparities is integral to ensuring interventions do not widen existing inequities. If interventions are found to provide less benefit to already disadvantaged populations, they can be adapted to reduce disparities (Veinot et al., 2018). Community-based participatory research, human-centered design, and formative evaluation can be used to identify and address factors influencing the effectiveness of a potential intervention as well as develop and implement interventions customized for disadvantaged populations. In addition to the statistical limitations explored here, equity-motivated HTE analyses must consider other methodological challenges including: how to define and collect standard data on race and other social determinants of health (Williams et al., 1994; Krieger, 2000; Kaplan & Bennett, 2003); how to measure structural racism and other institutionalized forms of disadvantage (Adkins-Jackson et al., 2022; Hardeman et al., 2022); how to balance potential intervention HTEs against baseline differences in event and exposure rates (Ward et al., 2019); and how to disseminate and implement findings in a way that does not perpetuate stigma against populations with worse health outcomes (Boyd et al., 2020).

Overall, caution should be used when interpreting findings from a HTE analysis of cluster-level outcome by a characteristic defined at the individual level (e.g., self-reported race) that is aggregated to the cluster level. While the interpretation of the cluster-level HTE analysis results are not as straightforward as those from the corresponding individual-level HTE analysis, it still may be scientifically relevant to conduct the cluster-level analysis if the requisite data are available. Importantly, though testing for HTE at the cluster level may have low power, there still may be meaningful (though not statistically significant) differences in the intervention effect observed across subpopulations which could be acted upon. If one decides to conduct such an analysis, power calculations should be done by simulating the individual-level data that is then aggregated rather than simulating the cluster-level data directly for both the characteristic of interest and outcome. One needs to truly capture the underlying variability of all variables included in the HTE analysis to understand the power limitations in this setting.

## Data Availability

All results may be reproduced using code available on GitHub at https://github.com/bdwilliamson/cluster_subgroup_analyses_supplementary.
