# Correction: Considerations for Subgroup Analyses in Cluster-Randomized Trials Based on Aggregated Individual-Level Predictors

**DOI:** 10.1007/s11121-024-01647-0

**Published:** 2024-01-15

**Authors:** Brian D. Williamson, R. Yates Coley, Clarissa Hsu, Courtney E. McCracken, Andrea J. Cook

**Affiliations:** 1 Biostatistics Division, Kaiser Permanente Washington Health Research Institute, Seattle, WA USA; 2 Vaccine and Infectious Disease Division, Fred Hutchinson Cancer Center, Seattle, WA USA; 3 Department of Biostatistics, University of Washington, Seattle, WA USA; 4 Investigative Science Division, Kaiser Permanente Washington Health Research Institute, Seattle, WA USA; 5 Center for Research and Evaluation, Kaiser Permanente Georgia, Atlanta, GA USA

**Correction to: Prevention Science** 10.1007/s11121-023-01606-1

The article “Considerations for Subgroup Analyses in Cluster-Randomized Trials Based on Aggregated Individual-Level Predictors”, written by Williamson, B.D., Coley, R.Y., Hsu, C., McCracken, C.E., and Cook, A.J., was originally published electronically on the publisher’s internet portal on 28 October 2023 without open access. With the author(s)’ decision to opt for Open Choice the copyright of the article changed on 26 December 2023 to © The Author(s) 2023 and the article is forthwith distributed under a Creative Commons Attribution 4.0 International License, which permits use, sharing, adaptation, distribution and reproduction in any medium or format, as long as you give appropriate credit to the original author(s) and the source, provide a link to the Creative Commons licence, and indicate if changes were made. The images or other third party material in this article are included in the article’s Creative Commons licence, unless indicated otherwise in a credit line to the material. If material is not included in the article’s Creative Commons licence and your intended use is not permitted by statutory regulation or exceeds the permitted use, you will need to obtain permission directly from the copyright holder. To view a copy of this licence, visit http://creativecommons.org/licenses/by/4.0/.

The original article has been corrected.

